# Stay in Touch—The Cortical ER of Moss Protonemata in Osmotic Stress Situations

**DOI:** 10.3390/plants9040421

**Published:** 2020-03-30

**Authors:** Dominik Harant, Ingeborg Lang

**Affiliations:** 1 Core Facility Cell Imaging & Ultrastructure Research, Faculty of Life Sciences, The University of Vienna, Althanstrasse 14, A-1090 Vienna, Austria; dominik.harant@hotmail.com; 2 Department of Functional and Evolutionary Ecology, Faculty of Life Sciences, The University of Vienna, Althanstrasse 14, A-1090 Vienna, Austria

**Keywords:** osmotic stress, cortical ER, actin, latrunculin B, *Physcomitrella patens*, moss, plasmolysis

## Abstract

Plasmolysis is usually introduced to cell biology students as a tool to illustrate the plasma membrane: hypertonic solutions cause the living protoplast to shrink by osmotic water loss; hence, it detaches from the surrounding cell wall. What happens, however, with the subcellular structures in the cell cortex during this process of turgor loss? Here, we investigated the cortical endoplasmic reticulum (ER) in moss protonema cells of *Physcomitrella patens* in a cell line carrying a transgenic ER marker (GFP-HDEL). The plasma membrane was labelled simultaneously with the fluorescent dye FM4-64 to achieve structural separation. By placing the protonemata in a hypertonic mannitol solution (0.8 M), we were able to follow the behaviour of the cortical ER and the protoplast during plasmolysis by confocal laser scanning microscopy (CLSM). The protoplast shape and structural changes of the ER were further examined after depolymerisation of actin microfilaments with latrunculin B (1 µM). In its natural state, the cortical ER is a dynamic network of fine tubes and cisternae underneath the plasma membrane. Under acute and long-term plasmolysis (up to 45 min), changes in the protoplast form and the cortical ER, as well as the formation of Hechtian strands and Hechtian reticula, were observed. The processing of the high-resolution z-scans allowed the creation of 3D models and gave detailed insight into the ER of living protonema cells before, during and after plasmolysis.

## 1. Introduction

Bryophytes were among the first plants to settle on dry land and even today, bryophytes play an essential role as pioneers in colonising new areas by forming substrate and seed beds for vascular plants [1]. The dominant haploid gametophyte consists of the gametophore, containing leaflets and stems, and the protonema, filamentous threads of cells showing tip growth and eventual branching [2]. Firstly, protonema cells form chloronema with orthogonal cell walls and relatively large, cylindrical cells containing many chloroplasts [3,4]. In later development, the protonema forms branches and buds of gametophores as well as caulonema cells with thinner and longer shapes [5]; caulonema cell walls are oblique and the cells contain less and differently shaped chloroplasts [4].

Among bryophytes, *Physcomitrella patens* is commonly used as a model organism for mosses. Various cell lines are available with GFP-tagged proteins and due to its rapid growth, abundance and long, threaded, cylindrical cells, *P. patens* protonemata represent an excellent model to study the physiological effects of cells, organelles, or subcellular structures and their organisation, e.g., under osmotic stress.

Plant cells use osmoregulation to withstand natural variations of water loss from the vacuole in drought situations. We can simulate water loss by placing plant cells in hypertonic solutions. If the hypertonic solution exceeds in the osmotic value of the cell, the protoplast shrinks due to the water loss leading to plasmolysis. In this reversible process, the living protoplast becomes detached from its surrounding cell wall [6]. While the volume of the protoplast decreases in plasmolysis, the surplus plasma membrane is endocytosed or remains partly anchored to the cell wall by forming Hechtian strands and reticula in the plasmolytic space [7,8,9].

Thus, plasmolysis is an excellent tool to investigate these membrane–wall contacts. Moreover, the osmotic stress during plasmolysis allows further analyses on the physiological and structural changes of subcellular compartments, such as the endoplasmic reticulum (ER), or the cytoskeleton [10].

As part of the endomembrane system, the ER is known to play an essential role in protein, membrane and lipid synthesis. The ER is connected to dictyosomes, the vacuole, the mitochondria, chloroplasts, plasmodesmata and the plasma membrane via different domains [11]. The cortical ER is located directly beneath the plasma membrane. It branches in multiple three-way junctions resulting in a complex network of fine tubules and cisternae. Reticulon proteins are responsible for its tubular and areal appearance [12,13] and the ER network is constantly changing by forming new branches and tubes which lead to versatile polygons [14,15]. Persistency-maps in tobacco leaves have shown that the majority of the network underlies constant movement and change [16]. Only 5% of the ER is fairly immobile, with ER–plasma membrane contact sites (ERPCs) forming possible anchor points, fixed by microtubules [17,18,19]. Dynamic actin filaments are responsible for the constant shifts of shape of the ER network [11]. 

Here, we used plasmolysis as a tool to induce forced, structural changes of the cortical ER, due to the shrinkage of the protoplast in water loss, to observe the behaviour of the ER network after the destruction of the stabilising actin filaments during this process.

## 2. Materials and Methods 

### 2.1. Plant Culture 

*Physcomitrella patens* (Funariaceae) was grown in sterile tissue culture on a solid PpNH_4_-moss medium, containing MgSO_4_∙7H_2_O, KH_2_PO_4_, CaNO_3_·4H_2_O, FeSO_4_·7H_2_O, Di-Ammoniumtartrate and micro-elements (https://sites.dartmouth.edu/bezanillalab). Subculturing was performed every 5 weeks by the inoculation of small gametophore parts into new agar plates. Moss plates were kept in a culture cabinet at 20 °C with a 14 h light/10 h dark cycle and 50% relative humidity. For experiments and microscopy, chloronema and caulonema cells were transferred to a glass slide and placed between two stripes of vaseline (applied with a brush while molten). The sample was then sealed with a cover slip. These chambers ensured that the protonemata were not squeezed by the weight of the coverslip and that various liquids for consecutive experiments could be easily applied through the vaseline channel.

### 2.2. Fluorescence Markers and Staining

For the visualisation of the ER, we used a *P. patens* cell line expressing mGFP::ER::HDEL. The labelling of the plasma membrane and eventually, the Hechtian strands and reticula occurring in plasmolysis, was performed by the application of 36 µM FM4-64 (ThermoFisher Scientific, Waltham, MA, USA) for 60 min. The cells were washed 3 to 4 times in 100 µL distilled water prior to subsequent treatments with solutions for plasmolysis or actin depolymerisation (see below). Chloroplasts were detected due to their chlorophyll fluorescence.

### 2.3. Reduced Turgor and Plasmolysis

Protonema cells were plasmolysed by treatment with a 0.8 M mannitol solution. To transfer the cells to the plasmolysis solution, 100–150 µL mannitol solution was soaked through the vaseline channel. During microscopic observation, the ends of the vaseline channels were always covered by a mannitol film to counteract variations of the solution’s osmolarity. The behaviour of the plasma membrane, the ER and actin filaments in the first 30 min of mannitol treatment, so-called acute plasmolysis, as well as long-term plasmolysis (30 to 45 min) was examined.

### 2.4. Actin Depolymerisation

To observe the possible changes in the ER structure related to the actin cytoskeleton, we treated the cells with the actin depolymerisation drug latrunculin B (LatB; Sigma Aldrich, St. Louis, MO, USA). LatB has been frequently used in plant cells, including mosses [20,21]. A 1 µM solution was applied through the vaseline channel. Observations took place after 30 min of incubation at room temperature. FM4-64-staining for the plasma membrane occurred prior to LatB treatment; eventual plasmolysis experiments with mannitol solution were done after LatB application.

### 2.5. Confocal Laser Scanning Microscopy (CLSM)

All the observations were performed on living cells with an upright confocal laser scanning microscope (Leica TCS SP5 DM-6000 CS, Leica Microsystems, Vienna, Austria) and LAS AF Software v4 (Leica Microsystems, Vienna, Austria). The images were obtained by a 63× water immersion objective (NA 1.2). For the excitation of the different fluorophores, we used a multi-argon laser and selected the wavelength of 488 nm for excitation. Three different photomultipliers simultaneously detected the emission wavelengths of GFP (495–640 nm), FM4-64 (575–640 nm), and chlorophyll (670–770 nm), respectively. The pinhole was set to one airy disc and the focal depth was about 0.5 µm. The scanning speed was set between 200 and 400 Hz. For observations of acute plasmolysis, it was increased to 700 Hz. Z-stacks of 20–300 images were used as maximum projection or videos, and single images were processed and further investigated with FIJI software [22].

### 2.6. Three-Dimensional Reconstruction

For high-resolution 3D images of the protonema cells, the step size in z-direction was set to “system-optimised“ (0.13 µm) which is equivalent to the resolution in the xy direction. The resulting z-stacks contained approximately 100–300 optical sections and were transferred to the 3D visualisation program AMIRA^®^ 6.2.0 (FEI). After segmentation with the threshold tool of the labelled membrane, ER and chloroplasts, we generated a smoothed and pseudo-coloured surface to further examine the various membrane structures.

## 3. Results

### 3.1. The Cortical ER of P. patens Before Plasmolysis

**Figure 1:**
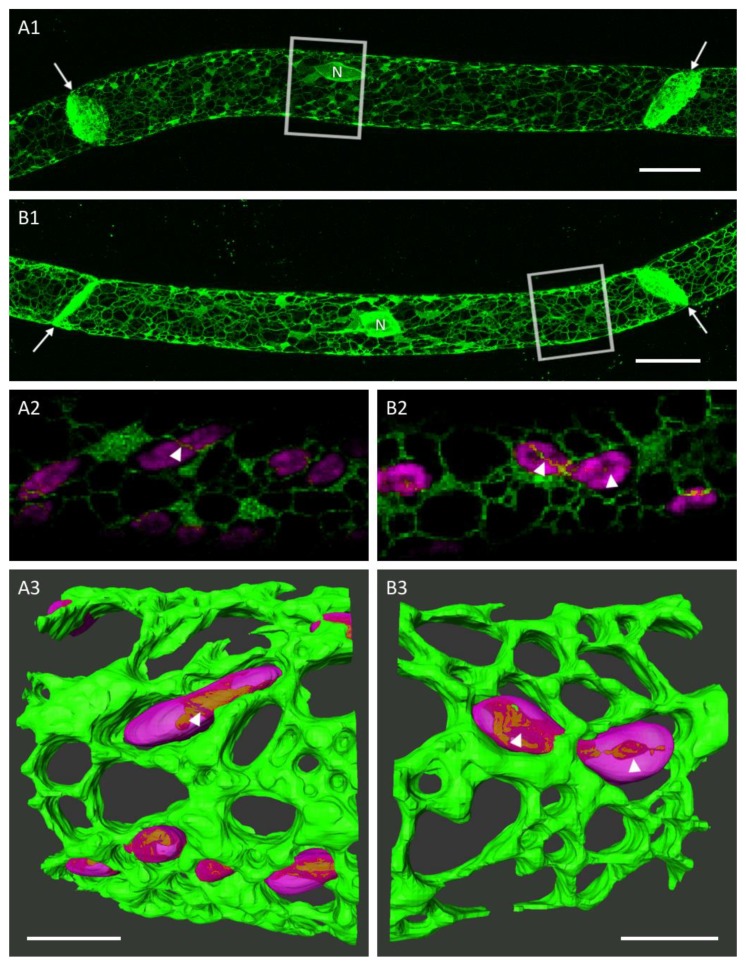
Two caulonema cells with a cortical endoplasmic reticulum (ER) network of fine tubules and areal cisternae. **A1**, **B1**: Maximum projections show a high abundance of ER near the nucleus (N) and the connecting cell wall (arrows). Scale bars 20 µm. **A2**, **B2**: Tangential 3D slices of A1 and B1 show the ER tubules and sheets in very close vicinity to chloroplasts (arrowheads). **A3**, **B3**: Three-dimensional-surface-rendering of the regions indicated by the boxes in A1 and B1. Chloroplasts were set transparent to show seemingly piercing ER tubules (arrowheads). Scale bars 5 µm.

**Figure 2:**
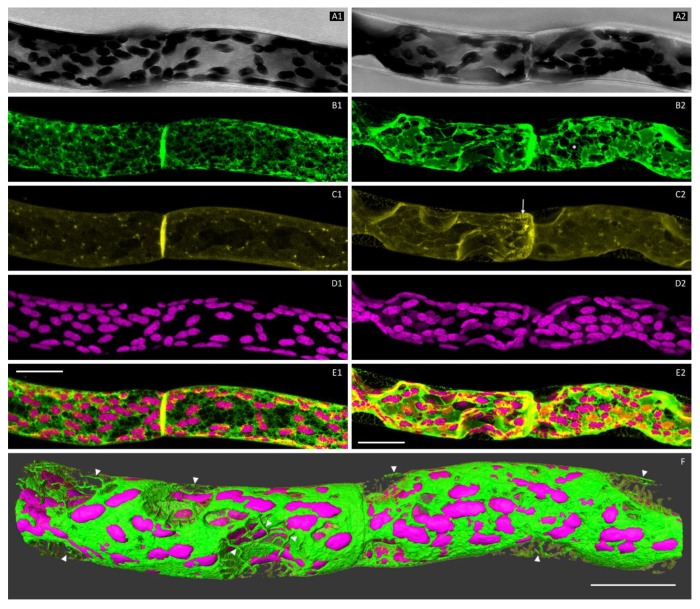
Maximum projections of the chloronema cells before (**A1**–**E1**) and after plasmolysis (**A2**–**E2**, **F**). **A1**, **A2**: Brightfield-Image. **B1**, **B2**: ER labelled by GFP-HDEL. **C1**, **C2**: Plasma membrane and endocytotic vesicles (arrow in C2) stained with FM4-64. **D1**, **D2**: Autofluorescence of the chlorophyll in the chloroplasts. **E1**, **E2**: Merged image of all detected fluorescence channels. **F**: Three-dimensional reconstruction of the plasmolysed cells shown in A2–E2. ER aggregated to bigger sheets interconnected via fine tubes, especially near chloroplasts (asterisk in B2). Concave areas formed at the detachment site of the protoplast from the cell wall due to the osmotic water loss. The plasma membrane forms a Hechtian reticula, sometimes also containing ER (arrowheads). Scale bars 20 µm. Supplemental Video S1.

The cortical ER of caulonema cells consists of a dense network in the cell cortex. Fine tubules form polygons of different sizes interconnected by three-way junctions and leaving gaps for chloroplasts. ER tubes also connect the relatively small areal cisternae which are primarily detected near chloroplasts and the nucleus (Figure 1A1,B1 and Figure 2A1–E1). The 3D reconstructions and tangential slices of the images show fine ER tubules in close vicinity to individual chloroplasts and the connection of small chloroplasts enclosed by ER sheets (Figure 1A2,A3,B2,B3).

### 3.2. Plasmolysis of P. patens Protonema Cells

**Figure 3:**
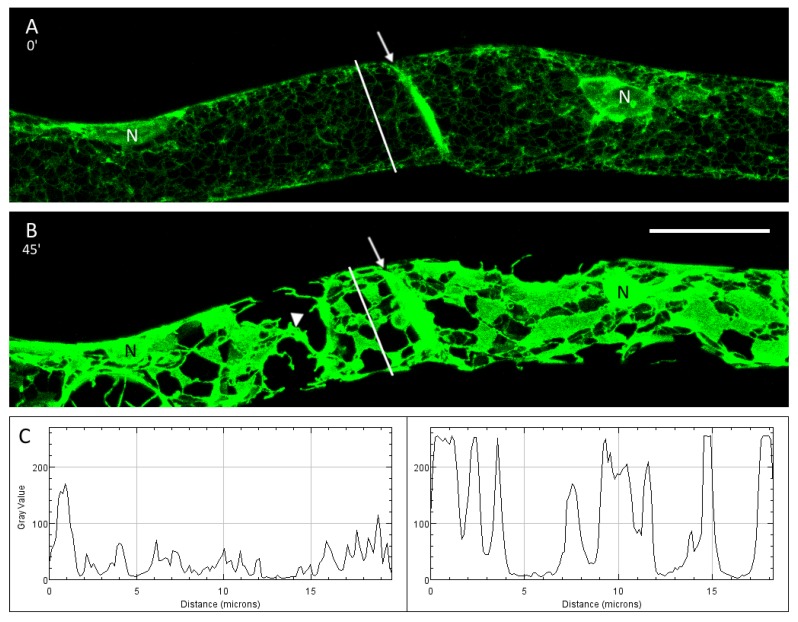
The ER network before (**A**) and after plasmolysis (**B**; 45 min in mannitol). The ER aggregates into big sheets leaving large holes in the cortical ER network. In the left cell, the two sub-protoplasts are connected via a branch of Hechtian reticulum (arrowhead). At the connecting cell wall, very bright fluorescence was detected (arrow). **C**: Fluorescence intensity values of GFP-HDEL along the cross-section (white line in A and B, respectively) show great differences before and after plasmolysis. Scale bar 20 µm.

**Figure 4:**
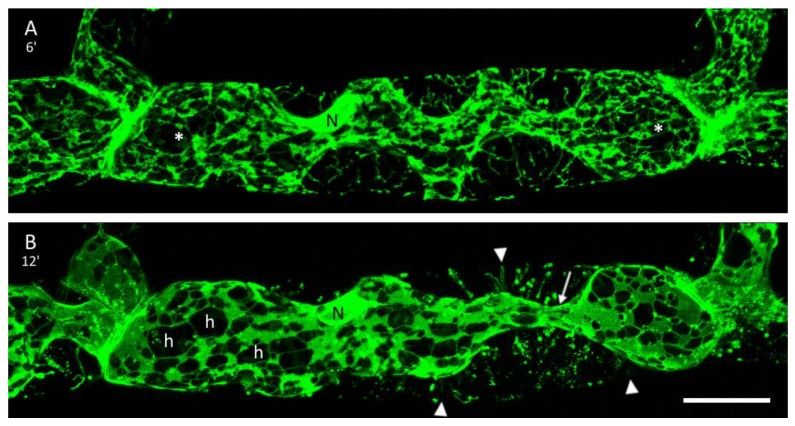
Dynamic adjustments: **A**: Six minutes in mannitol solution caused concave detachments and Hechtian reticula containing ER. Concave detachments at the top of the cell were marked with asterisks. **B**: The same cell after 12 min in mannitol. Some of the big membrane–wall connections are lost (arrowheads), resulting in convex protoplasts connected by a cytoplasmic strand (arrow). Parts of the Hechtian reticula were disrupted during this process. The ER merges into larger sheets with interconnected tubules and large holes (h) in the cortex. Scale bar 20 µm.

**Figure 5:**
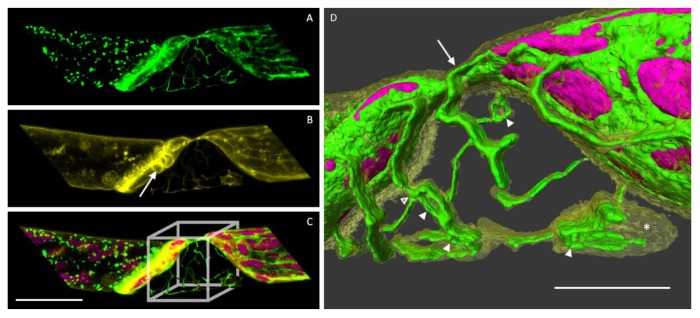
ER in Hechtian reticula after 30 min mannitol treatment (left cell died during this experiment). **A**: GFP-ER signal. **B**: FM4-64-labelled plasma membrane and osmocytotic vesicles (arrow). **C**: Merge of all detected channels including the autofluorescence of chloroplasts. Scale bar 20 µm. **D**: Three-dimensional reconstruction of the region of interest in C (box). Scale bar 10 µm. The ER forms branches and rings (arrowheads) inside the Hechtian reticula. The empty arrowhead points to a polygon in the background. The two protoplasts portions at the cell wall remain connected via branching Hechtian reticula (arrow). The asterisk marks the Hechtian reticula area without ER. Supplemental Video S2.

After staining the plasma membrane with FM4-64, the cells were first scanned in their natural state (Figure 2A1–E1). The regions of interest were scanned in the z direction for the image stacks of high resolution resulting in maximum projections and 3D reconstructions (supplemental Video S1). Subsequently, the cells were placed into a hypertonic mannitol solution (0.8 M). At first, osmotic water loss caused concave detachments of the plasma membrane from lateral cell walls. Hechtian reticula appeared at the detachment sites, containing ER (Figure 2A2–E2,F). As plasmolysis proceeded, the protoplasts curved into a more convex shape, often resulting in sub-protoplasts connected via a cytoplasmic strand (Figure 3B and Figure 4B; supplemental Figure S1). In the shrinking protoplasts, a progressive aggregation of the ER network, especially around the chloroplasts, was observed. The FM4-64-labelled plasma membrane was endocytosed during plasmolysis (osmocytosis), leading to vesicles of different sizes (Figure 2C2 and Figure 5B).

### 3.3. The ER and Plasmolysis

Before plasmolysis the cortical ER was observed as a network of fine tubules (Figure 3A). In the hypertonic environment, thin tubules aggregated progressively into larger sheets and areal cisternae resulting in bigger holes and gaps at the cell cortex (Figure 3B and Figure 4A,B). A closer look at the maximum projections of cells before and after mannitol treatment revealed changes in the fluorescence intensity of the GFP-tagged ER (Figure 3C). Although the fluorescence intensities varied widely in control cells, the values were relatively low. After plasmolysis, the GFP signal intensity increased substantially and peak values were consistently high. The graphs also show the merging of membranes into bigger aggregates and sheets. Very bright ER fluorescence was also detected at the connecting cell wall (Figure 3A,B). The membrane–wall connections can become disrupted leading to drastic changes of the protoplast form within a few minutes (Figure 4A,B).

### 3.4. The ER in Hechtian Reticula

Branched membrane strands, Hechtian reticula, formed at the onset of plasmolysis and remained attached to the cell wall (Figure 2F). As 3D reconstructions in higher magnification revealed, ER was detected in most of the Hechtian reticula as it forms branches, polygons and rings therein (Figure 5A–D, supplemental Video S2). However, not all the Hechtian reticula were fully filled with ER (Figure 5D). With the progressive osmotic reduction of the protoplast, the aggregated ER network of two separated sub-protoplasts remained strongly connected by Hechtian reticula (Figure 5D) or cytoplasmic strands (Figure 4B). 

### 3.5. ER after Treatment with Latrunculin B 

**Figure 6:**
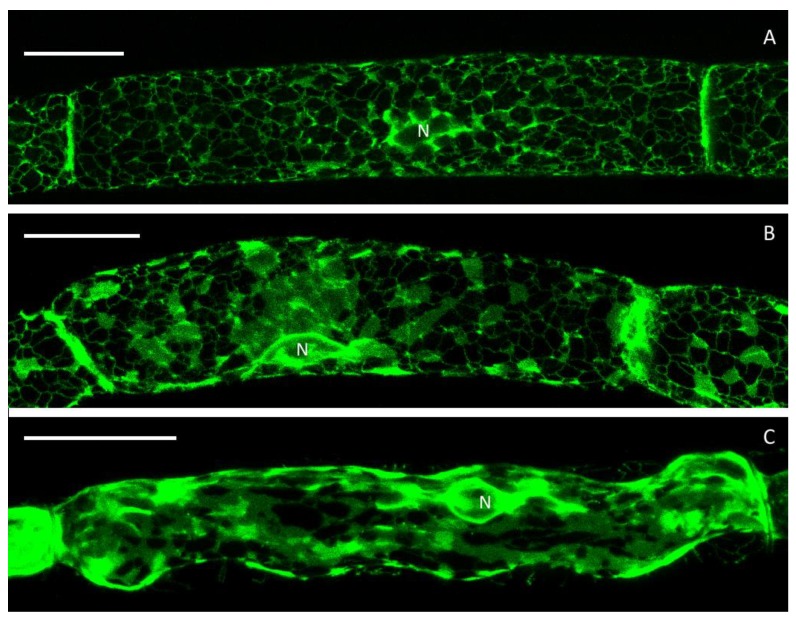
The cortical ER network in control cells before (**A**) and after actin depolymerisation with LatB (**B**). **C** shows the aggregated ER sheets after a combination of LatB treatment and plasmolysis. Scale bars 20 µm.

To investigate the role of actin in stabilizing and shaping the ER network, protonema cells were treated with 1 µM latrunculin B (LatB) to depolarize actin filaments (Supplemental Figure S1). Before LatB treatment, the ER showed the regular, fine and tightly meshed tubular network with only small cisternae (Figure 6A). After LatB application for 30 min, however, many ER tubules disintegrated into wider sheets (Figure 6B) as in plasmolysed cells (Figure 4B). Furthermore, distinct three-way junctions were lost and almost completely disappeared after a combination of LatB treatment and plasmolysis (Figure 6C).

## 4. Discussion 

The cortical ER of plant cells consists of a network of tubules and sheets [11,23,24,25]. Single tubules can form polygons of varying diameters and are connected by three-way junctions [26]. In *Physcomitrella patens*, the differentiation of protonema cells into caulonema and chloronema is fluent and we might have imaged different developmental stages of cells as in caulonema cells of the moss *Funaria hygrometrica*, where denser ER portions were reported in young parts of the cells [23]. Super-resolution imaging techniques in animal cells revealed that areal parts of the ER may consist of highly condensed tubules instead of sheets [27]. The high dynamic of ER structures and confocal microscopy as applied here did not provide the necessary resolution in time to answer this question for plant ER. Segmentation and 3D reconstructions, however, allowed for a very detailed look at the cortical ER and its close connection with cell organelles like chloroplasts (Figure 1) that would need to be further investigated with molecular methods. The power of this connection was impressively demonstrated by laser tweezers; even a vigorous mechanical displacement of chloroplasts could not detach it from a connecting ER tubule [28].

Reticulon proteins play a major role in the transition from sheets into tubules within the ER. Their incorporation into the lipid bilayer results in a membrane curvature [13,29]. Consecutively, reticulons have been shown along ER tubules but also at the edges of ER sheets and holes [30,31]. Here, we also observed a transition from ER tubules into sheets in plasmolysis (Figure 3), as also shown by fluorescence intensity maps in Figure 3C. In parts, a merging of membranes in the shrunken protoplast is explained by the adjustment to a smaller space thereby forming more sheets. Interestingly, treatment with LatB, a drug to depolymerize actin microfilaments, resulted in a similar formation of ER sheets in the protoplast (Figure 6) like during plasmolysis. Hence, actin microfilaments are closely linked to the cortical ER and play a crucial role in its shaping, as previously reported [32]. A similar, stabilising effect of actin microfibrils and bundles thereof has been reported for plasmolysed protoplasts of *Chlorophyton comosum* [10] and we see similar structures in strands between sub-protoplasts (Supplemental Figure S1). Consistency maps by Cheng et al. ([24]) further confirmed the supportive importance of actin in shaping the ER particularly in Hechtian strands. Interestingly, the Hechtian reticulum contained less polymerised actin but more bundled microtubules [24].

The activity of many actin-binding proteins is calcium-dependent. Hence, calcium plays a direct role in the modulation of F-actin architecture. Recently developed genetically encoded calcium indicator probes allow to trace calcium modulations in vivo via FRET (Förster Energy Resonance Transfer) or GFP-linked techniques. At their core, each sensor contains an engineered calcium binding calmodulin variant and calmodulin-binding peptide fused to one or two fluorophores that optically report Ca^2+^-dependent conformational changes [33]. Bascom et al. [21]. applied such probes to tip growing *P. patens* chloronema cells and revealed cytosolic calcium amounts to be directly linked to actin polymerisation and depolymerisation, respectively. Oscillatory changes in calcium precisely modulated the apical actin spot, thereby leading to the regulation of cell expansion at the cell tip [21]. Extracellular calcium influx, mediated by the plasma membrane, could be inhibited by LaCl3, a blocker of non-selective calcium channels [21] but its further transport to the ER, organelles or other compartments remains unclear. Interestingly, the amount and dynamics of calcium in the ER were substantially different to cytosolic ones in *Arabidopsis* root tip cells [34]. Various pharmacological tests using the abovementioned calcium indicator probes revealed little contribution of the ER in calcium storage. In contrast, the amount of calcium in the ER always followed cytosolic concentrations in the tested *Arabidopsis* root cells [34].

In the perception of external stimuli such as osmotic stress by dehydration, calcium signals have been reported as cell type and tissue-specific waves [35]. Although *P. patens* is lacking a proper vascular tissue, the calcium waves expanding from the base to the tip of the gametophore suggest the different calcium sensitivity of specific cells.

In plasmolysis, the dramatic osmotic water loss causes the separation of the plasma membrane from the cell wall in protonema cells. The underlying cytoplasm including the ER is following the shape of the protoplast. In *P. patens* protonema cells, concave detachment sites are formed at first and a network-like structure, the Hechtian reticulum [7], is left at the cell wall. Three-dimensional segmentation and rotation of the Hechtian reticulum clearly showed its composition of both, ER and plasma membrane (Figure 5) as also reported by Cheng et al. [24] for seed plants. Within the cytoplasmic portions left at the cell wall, the ER formed polygons and sheets with occasional branches but not all portions covered by plasma membrane were filled with ER tubules (Figure 5). 

There is still a debate on the attachment sites of the plasma membrane to the cell wall in plasmolysis. The similarity of ER tubules in Hechtian reticula and desmotubules resulted in assumptions that plasmodesmata provide these attachment points. In addition, reticulons are important for primary plasmodesmata formation [36]. However, *P. patens* protonemata consist of lines of single cells with only one connecting cell wall between neighbours. No plasmodesmata are built towards the side of the cells along the longitudinal axis and yet, Hechtian reticula are very prominent at these cell walls. In animal cells, ER–plasma membrane contact sites (ECPS) are essential for the maintenance and regulation of neuronal processes in dendrites, providing lipid transfer, signalling, Ca^2+^ homeostasis and synaptic plasticity [37]. Similarly, EPCS with potential connections to the cell wall have been described in plant cells [17]. A comprehensive review on the plasma membrane–cell wall continuum is given by Liu et al. [38]) showing various options for physical links of the cell wall to the plasma membrane although still many questions remain. 

Persistent plasmolysis for more than 10 min resulted in the transition from concave detachments into more convex protoplast shapes (Figure 4). Osmocytotic vesicles to reduce membrane material in plasmolysis [39,40] become visible as shown in Figure 5B. Multiple waves of plasmolysis in differentially labelled osmotic solutions showed separated populations of osmocytotic vesicles [41]. Here, we did not follow the fate of these vesicles in long-term plasmolysis nor their potential inclusion in the ER and other degradation processes.

## 5. Conclusions

Although mosses are evolutionary separate from seed plants, the structure of the cortical ER is almost identical in its appearance and behaviour in both, control and osmotically stressed cells. Plasmolysis caused a transition from tubular ER networks to sheets. Depolymerisation of actin microfilaments has a similar effect on the cortical ER pointing to changes in the actin cytoskeleton during plasmolysis. Not only the ER but also the plasmolysis forms change over time. Segmentation and 3D reconstruction allowed the investigations of subcellular compartments and the distinct localization of ER in *Hechtian reticula*.

## Supplementary Materials

The following are available online at https://www.mdpi.com/2223-7747/9/4/421/s1. Supplemental Video S1: Three-dimensional reconstruction of two protonema cells of *P. patens* after osmotic water loss in plasmolysis; Supplemental Video S2: A close-up of Hechtian reticulum showing the distinct separation of ER (green) and plasma membrane (yellow). Supplemental Figure S1: P. patens cell line expressing GFP::Actin after plasmolysis. (**A**) the protonema cells show filamentous actin and the subprotoplasts remain connected by strands with bright actin content (arrows). (**B**) after 30 min treatment with 1 µM LatB, actin filaments were destroyed and non-specific GFP-fluorescence appeared in the cytoplasm and around the chloroplasts (**B**). Scale bars 20 µm.
