# Supplementary material for: Stay in Touch—The Cortical ER of Moss Protonemata in Osmotic Stress Situations
Source: Plants (Basel). 2020 Mar 30;9(4):421. doi: 10.3390/plants9040421 (PMC7238208; doi:10.3390/plants9040421)
Supplement: Supplementary file 1 [file plants-09-00421-s001.zip › plants-744846-supplementary_final/plants-744846_Suplementary_Figure_final.pdf]

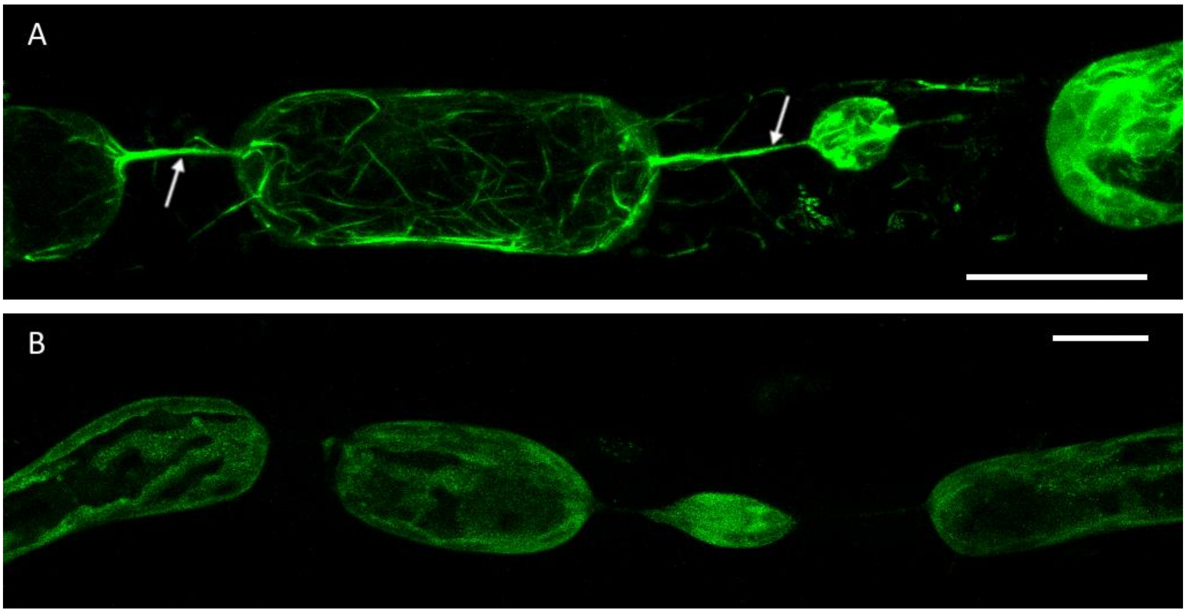

Supplemental Figure 1. *P. patens* cell line expressing GFP::Actin after plasmolysis. **A**) the protonema cells show filamentous actin and the subprotoplasts remain connected by strands with bright actin content (arrows). **B**) after 30 min treatment with 1 µM LatB,, actin filaments were destroyed and non-specific GFP-fluorescence appeared in the cytoplasm and around the chloroplasts (**B**). Scale bars 20µm.
